# Transcriptional Analysis of C-Repeat Binding Factors in Fruit of *Citrus* Species with Differential Sensitivity to Chilling Injury during Postharvest Storage

**DOI:** 10.3390/ijms22020804

**Published:** 2021-01-15

**Authors:** Matías Salvo, Florencia Rey, Ana Arruabarrena, Giuliana Gambetta, María J. Rodrigo, Lorenzo Zacarías, Joanna Lado

**Affiliations:** 1 Programa de Investigación en Citricultura, Estación Experimental INIA Salto Grande, Instituto Nacional de Investigación Agropecuaria (INIA), Camino a la Represa s/n, 50000 Salto, Uruguay; matiassalvo28@gmail.com (M.S.); aarruabarrena@inia.org.uy (A.A.); 2 Food Biotechnology Department, Instituto de Agroquímica y Tecnología de Alimentos, Consejo Superior de Investigaciones Científicas (IATA-CSIC), Paterna, 46980 Valencia, Spain; floreyrob@iata.csic.es (F.R.); mjrodrigo@iata.csic.es (M.J.R.); lzacarias@iata.csic.es (L.Z.); 3 Departamento de Producción Vegetal, Facultad de Agronomía, Universidad de la República, Garzón 780, 11900 Montevideo, Uruguay; gambetta@fagro.edu.uy

**Keywords:** CBF, citrus, chilling injury, DREB, gene expression

## Abstract

Citrus fruit are sensitive to chilling injury (CI) during cold storage, a peel disorder that causes economic losses. C-repeat binding factors (CBFs) are related to cold acclimation and tolerance in different plants. To explore the role of *Citrus* CBFs in fruit response to cold, an in silico study was performed, revealing three genes (*CBF1*, *CBF2*, and *CBF3*) whose expression in CI sensitive and tolerant cultivars was followed. Major changes occurred at the early stages of cold exposure (1–5 d). Interestingly, *CBF1* was the most stimulated gene in the peel of CI-tolerant cultivars (Lisbon lemon, Star Ruby grapefruit, and Navelina orange), remaining unaltered in sensitive cultivars (Meyer lemon, Marsh grapefruit, and Salustiana orange). Results suggest a positive association of *CBF1* expression with cold tolerance in *Citrus* cultivars (except for mandarins), whereas the expression of *CBF2* or *CBF3* genes did not reveal a clear relationship with the susceptibility to CI. Light avoidance during fruit growth reduced postharvest CI in most sensitive cultivars, associated with a rapid and transient enhance in the expression of the three CBFs. Results suggest that CBFs-dependent pathways mediate at least part of the cold tolerance responses in sensitive *Citrus*, indicating that *CBF1* participates in the natural tolerance to CI.

## 1. Introduction

*Citrus* is one of the most important fruit crops worldwide, commercialized as fresh fruit or concentrated juice. Export of fresh citrus fruit to certain international markets requires quarantine cold treatments to avoid fruit fly [1]. However, cold storage (0–1 °C) during long transport could exert negative effects on the fruit of citrus cultivars sensitive to cold. Damage induced by low temperature, known as chilling injury (CI), is usually manifested in the peel, affecting the fruit’s external appearance and commercial quality [2]. Most characteristic symptoms of postharvest CI in the flavedo (external colored layer of the peel) are manifested as small, depressed areas that progressively become darker and sunken, producing large spots of brown or black color along the fruit surface [2,3].

The incidence of CI in citrus fruit depends on the species, the cultivar, growing conditions, pre-harvest temperatures, as well as fruit maturity at harvest [2,3]. Among the commercial *Citrus* species, limes, lemons, and grapefruit are considered highly sensitive to CI, more than the fruit of oranges and mandarins. It has been reported that white ‘Marsh’ grapefruit is more sensitive to cold than red-colored ‘Ruby Red’ and ‘Rio Red’ [4], while ‘Navel’ and ‘Blanca’ oranges are considered more tolerant than the `Shamouti’ cultivar [2,5]. In grapefruit, earlier and later harvested fruit are described to be more sensitive to CI than mid-season fruit [4,6,7]. By contrast, the opposite pattern of seasonal sensitivity to CI was observed in the cold-sensitive ‘Fortune’ mandarin under Mediterranean conditions [8], revealing that different pre-harvest factors may modulate fruit tolerance to cold storage. Moreover, we have previously observed that light deprivation in the red `Star Ruby` grapefruit induced resistance to CI, together with an increased lycopene content and singlet-oxygen antioxidant capacity, indicating the light exposure may directly or indirectly play a role in the tolerance of citrus fruit to CI [9,10,11].

Because of their subtropical origin, cold stress produces remarkable structural, biochemical, and molecular transformations in the peel of citrus fruit [2]. Changes in the expression of diverse genes related to a broad array of metabolic functions, such as stress stimuli, transcription factors, hormone biosynthesis, and carbohydrate metabolism, are stimulated or repressed by low temperatures [12,13,14]. Among transcription factors, C-Repeat Binding Factors (CBFs) have been described as relevant promoters of cold-tolerance associated responses in different cold-sensitive plant species [15], including citrus plants [16,17,18].

CBFs are transcription factors highly conserved among plants that bind to promoters of genes that respond to low temperatures (COR-cold regulated genes), stimulating their expression and participating in plant acclimation to and survival in low-temperature stress [19,20,21,22]. Products of the *COR* genes were suggested to be relevant in the acquisition of cold tolerance and include transcription factors, protein kinases, late embryogenesis abundant proteins, osmoprotectants, proteins associated with hormone responses, cell wall structure, and lipid metabolisms as well as chloroplastic proteins [23]. Expression of CBFs genes is stimulated a few hours or even minutes after tissue exposure to low temperatures in different plant organs [15,24]. Moreover, a high transcription of these genes induces cold tolerance in different species [15,25,26,27,28,29,30], including citrus plants [16,31].

In the model plant *Arabidopsis thaliana*, different CBFs genes have been reported, and their possible functions explored in relation to cold response and acclimation [21,32]. *CBF1* and *CBF3* are differentially regulated with respect to *CBF2*, while *CBF4* is not involved in response to low temperatures [15]. Indeed, the *cbf1*,*2*,*3* triple mutant showed an impaired freezing tolerance after cold exposition, establishing unequivocally that *CBF1*, *2*, and *3* genes are important regulators of cold acclimation in *Arabidopsis* [21]. Moreover, different ecotypes with contrasting sensitivity to cold exhibited clear differences in the expression of *CBF1* and *CBF2* genes [26]. Recent studies indicate that *COR* genes may also be regulated through CBF-independent pathways [23]. Evidence suggests that the three CBF proteins are partially redundant regulating *COR* genes, although some specialization has been inferred by differential expression patterns of these genes [33].

Plants integrate light and temperature signals to respond to changes in the environment. The expression of CBFs genes is also modulated by light in plants. The photoreceptor phytochrome B (phyB) was reported as responsible for the activation of cold-stress signaling in response to light. Light induces *CBF1*, *2*, and *3*, suggesting that there is a connection between cold and light signaling mediated by phytochromes in *Arabidopsis* [34]. Cold-induced CBFs proteins interact with phytochrome-interacting transcription 3 (PIF3) and phyB under cold stress in *Arabidopsis*, revealing that CBFs stabilize the phyB thermosensor to enhance plant freezing tolerance [35]. Studies in tomatoes revealed that *SlPIF4* directly binds to the promoters of *SlCBF* genes, and their expression is induced under low temperature via phytochrome A [36].

In *Citrus*, a possible role of CBFs in the differential cold tolerance of *Poncirus trifoliata* plants and pummelo (*Citrus grandis*) has been described since a lower gene expression was found in cold-sensitive pummelo than in *Poncirus* [31]. *PtCBF* expression was induced not only by low temperature but also by abscisic acid [17], a stress-response phytohormone. Similarly, differences in the cold-induced expression of *CBF1* between both species were observed since an earlier, and higher accumulation occurred in leaves of the cold-tolerant *Poncirus* compared to that of *C. paradisi* [16]. Moreover, *PtCBF1* putatively regulates *CORc115* expression (a cold-induced group II LEA gene) [16], which is part of the conserved plant responses to cold [23]. Therefore, CBFs appear to exert a role in the regulation of cold response in vegetative tissues of Citrus plants; however, information about the potential role of these transcription factors in the responses of fruit to cold during postharvest storage has not been yet addressed.

Transcriptional changes in *CBF* genes during fruit responses to low temperature have been explored in tomatoes, where the expression of *SlCBF1* is induced early by low temperatures and is associated with a higher tolerance to low temperatures [37]. Similarly, *CmCBF1* is induced after 6 and 12 h of cold storage, respectively, in the peel and pulp of melon fruit, with higher levels in the cold-tolerant cultivar [38]. In oil palm fruit (*Elaeis guineensis*), *EgCBF3* expression is induced after 2 h of cold treatment with a peak at 24 h [24]. In peach fruit, the transcription of *PpCBF1/5/6* is induced after 12 h of storage at 0 °C and is accompanied by a decrease in CI symptoms, whereas the expression of other *CBF* genes (*PpCBF2/3/4*) remains relatively constant [28]. Ectopic expression of a peach *PpCBF1* in apples increased freezing tolerance when compared to the non-transformed control [29]. Contrastingly, in table grapes, no induction in the expression of *VvCBF1* [39] and *VvCBF4/VviDREBA1–1* was observed during storage at 0 °C in the skin nor pulp of the fruit [40,41]. Current studies in *Citrus* suggest a role for CBFs in plant tolerance to cold under field conditions [16,31], but the involvement of CBFs in the cold tolerance of fruit during postharvest storage has not been explored. Therefore, the objective of this study was to investigate the potential role of *CBF* genes in the responses of citrus fruit to postharvest storage at low temperature. To unravel that goal, we used fruit of the main *Citrus* species (lemons, grapefruit, oranges, and mandarins) with contrasting susceptibility to develop CI during cold storage. Since the sensitivity of citrus fruit to CI can be influenced by pre-harvest conditions, such as light incidence during fruit growth [2], the effect of light deprivation in the expression of *CBF* genes in the peel of CI-susceptible fruit was also evaluated.

## 2. Results

### 2.1. In Silico Study of Citrus CBFs

**Figure 1:**
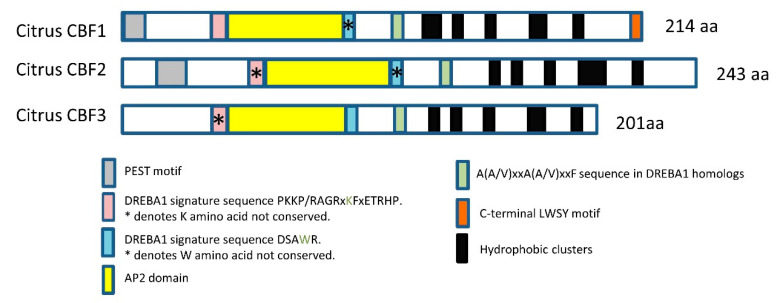
Schematic representation of C-repeat binding factors (CBFs) from *Citrus sinensis* (*CBF1*, orange1.1g028094m; *CBF2* orange1.1g026103m; *CBF3*, orange1.1g029015m) showing the main characteristics domains. The PEST motif (grey), the PKKPAGR (dehydration-responsive element binding (DREB) A 1 signature sequence PKKP/RAGRxKFxETRHP) motif (pink), the AP2 domain (yellow), the DSAWRL (DREBA1 signature sequence DS(A/V/S)WRL) motif (blue), and the A(A/V)xxA(A/V)xxF motif (green) are present in the citrus CBFs. The C-terminus hydrophobic clusters (HC2-HC6) (gray) are indicated. The C-terminal LWSY motif was only identified in citrus *CBF1*.

To identify all members of the CBFs family in *Citrus*, we first carried out a BLASTP search of the *Citrus sinensis* (sweet orange) genome database at Phytozome 12 (JGI, Sweet Orange Genome Project *Citrus sinensis* v1.1 https://phytozome.jgi.doe.gov/) with the *Arabidopsis* CBFs (*AtCBF1*; AT4G25490.1; *AtCBF2*, AT4G25470.1; *AtCBF3*, AT4G25480.1). *AtCBF4* was excluded from this analysis since it is involved in drought stress responses rather than cold [42]. The analysis revealed the presence of three genes encoding CBFs in *Citrus*: *CBF1* (orange1.1g028094m), *CBF2* (orange1.1g026103m), and *CBF3* (orange1.1g029015m). The length of predicted proteins was 214, 243, and 201 amino acids for *CBF1*, *2*, and *3*, respectively, and the C-terminal of all three proteins showed an acid isoelectric point as reported for dicot CBFs [40]. The search for functional and structural domains in the *Citrus* CBFs displayed most of the characteristic CBF features, although not all of them were fully conserved in all members (Figure 1). *CBF1* and *CBF2* showed the N-terminal PEST [peptide sequence rich in proline (P), glutamic acid (E), serine (S), and threonine (T)] domain (https://emboss.bioinformatics.nl/cgi-bin/emboss/epestfind), which is present in other members of the DREB (dehydration-responsive element binding) subfamily and has been associated with rapid protein turnover by targeting proteolytic degradation [40,43]. The CBF-conserved domains PKKRAGR (DREBA1 signature sequence PKKP/RAGRxKFxETRHP) and DSAWR (DREBA1 signature sequence DS(A/V/S)WRL) flanking the AP2 (Apetala2) domain were present in the three *Citrus* CBFs, but the full consensus sequences of both domains PKKRAGR and DSAWR were only conserved in *Citrus CBF1* and *3*, respectively (Figure 1 and Figure S1). The AP2 typical domain [44] showed a high degree of sequence identity with other plant CBFs (Figure 1 and Figure S1). The AP2 characteristic WLG and RAHD motifs, and valine (position14) and glutamic acid (position 19) were conserved in all citrus CBFs, but the YRG motif was only fully conserved in citrus *CBF1* (Figure S1). The AP2 downstream A(A/V)xxA(A/V)xxF sequence conserved in all DREBA1 homologs [45] was also identified in the three *Citrus CBF*, and the C-terminal LWSY motif [46] was only conserved in the *Citrus CBF1* (Figure 1 and Figure S1). The hydrophobic cluster analysis (HCA) of the C-terminus was performed (http://bioserv.rpbs.univ-paris-diderot.fr/services/HCA/) and showed that all *Citrus* CBFs contained five hydrophobic clusters, which has been described as important for trans-activation of target genes [47].

**Figure 2:**
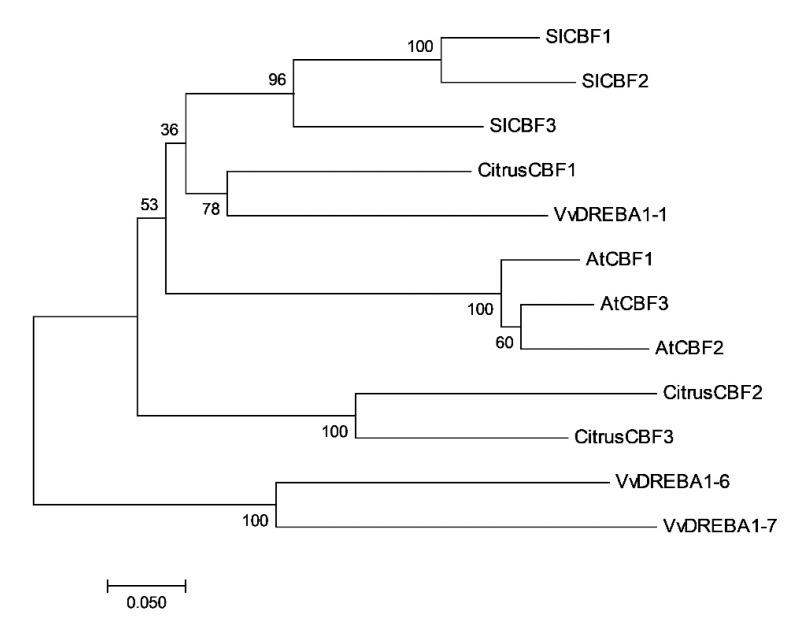
Phylogenetic tree of *Citrus* CBFs and other plant CBFs. The phylogenetic tree was generated based on the alignment of deduced amino acid sequences of *Citrus sinensis CBF1*, *2*, and *3* proteins and *Arabidopsis*, tomatoes, and table grapes CBFs. The tree was constructed based on the Neighbor-Joining method [48]. The percentage of replicate trees in which the associated taxa clustered together in the bootstrap test (1000 replicates) are shown next to the branches [49]. The tree is drawn to scale, with branch lengths in the same units as those of the evolutionary distances used to infer the phylogenetic tree. The sequences used to generate the phylogenetic tree and their accession numbers are as follows: *Citrus sinensis CBF1* (orange1.1g028094m), *CBF2* (orange1.1g026103m), and *CBF3* (orange1.1g029015m); *Arabidopsis thaliana AtCBF1* (AT4G25490.1), *AtCBF2* (AT4G25470.1) and *AtCBF3* (AT4G25480.1); *Solanum lycopersicum SlCBF1* (Q8S9N5), *SlCBF2* (XP_004234350.1) and *SlCBF3* (AAS77819.1); *Vitis vinifera VviDREBA1–6* (MF445008), *VviDREBA1–7* (MF445009) and *VviDREBA1–1* (MF445007). Evolutionary analysis was conducted in MEGA7 [50].

The comparison of the full protein sequences of *Citrus* CBFs revealed that *CBF2* and *3* were the most closely related sequences (72.8% identity), and both were least similar to *CBF1* (about 60% identity) (Table S1). The relationship between the *Citrus* CBFs with other plant CBFs proteins, including three members from *Arabidopsis*, tomatoes, and table grapes, was analyzed by sequence comparison and by the generation of a phylogenetic tree (Figure 2). The *Citrus CBF1* grouped in a cluster with table grape *VvDREBA1–1*, and this cluster was grouped with tomatoes and *Arabidopsis* CBFs (Figure 2). Interestingly, *Citrus CBF2* and 3 were located together in a separate branch and more distantly related to other CBFs (Figure 2). The comparison of full sequences of *Citrus* CBFs with other plant homologs showed that *Citrus CBF1* displayed a slightly higher percentage of identity with *Arabidopsis*, tomatoes, and table grape members, ranging from 49% to 67%, in comparison to *CBF2* and *CBF3* (43% to 62% of identity) (Table S2).

### 2.2. CI Symptoms and Expression of CBFs Genes in Cold-Tolerant and Cold-Sensitive Citrus Fruits during Cold Storage

**Figure 3:**
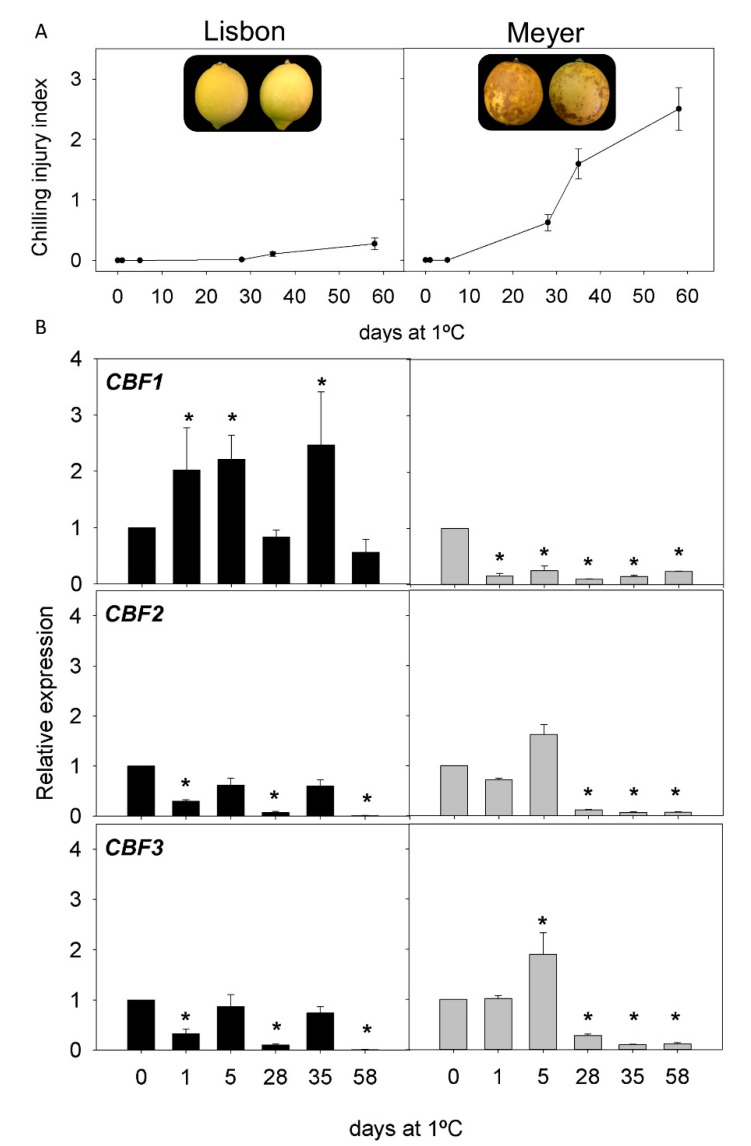
(**A**) Chilling injury (CI) index in Lisbon and Meyer lemons at harvest and during cold storage at 1 °C and (**B**) relative expression of *CBF1*, *CBF2*, and *CBF3* in Lisbon (black bars) and Meyer (grey bars) during cold storage (means ± S.E.). Pictures show the external appearance of fruit at 58 d of cold storage. For each cultivar, asterisks indicate significant differences in the expression of a *CBF* gene between each time-point and the harvest time (which were set to 1), by a Student’s *t*-test (*p* < 0.05).

**Figure 4:**
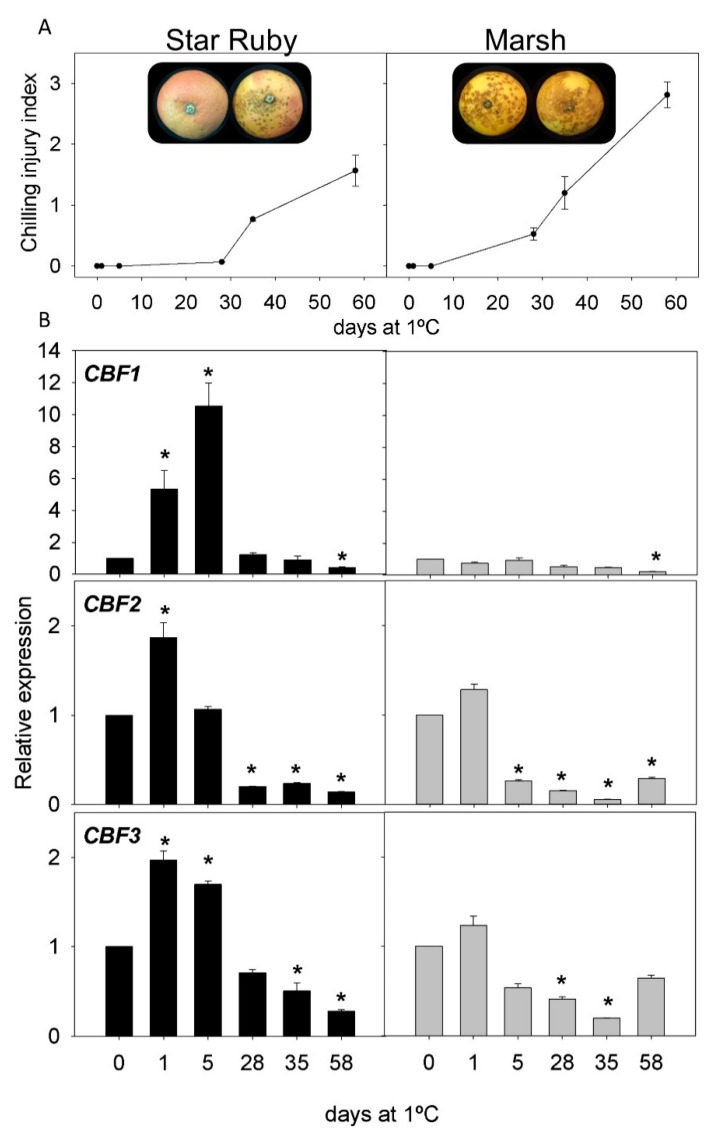
(**A**) CI index in Star Ruby and Marsh grapefruit at harvest and during cold storage at 1 °C and (**B**) relative expression of *CBF1*, *CBF2*, and *CBF3* in Star Ruby (black bars) and Marsh (grey bars) during cold storage (means ± S.E.). Pictures show the external appearance of fruit at 58 d of cold storage. For each cultivar, asterisks indicate significant differences in the expression of a *CBF* gene between each time-point and the harvest time (which were set to 1), by a Student’s *t*-test (*p* < 0.05).

**Figure 5:**
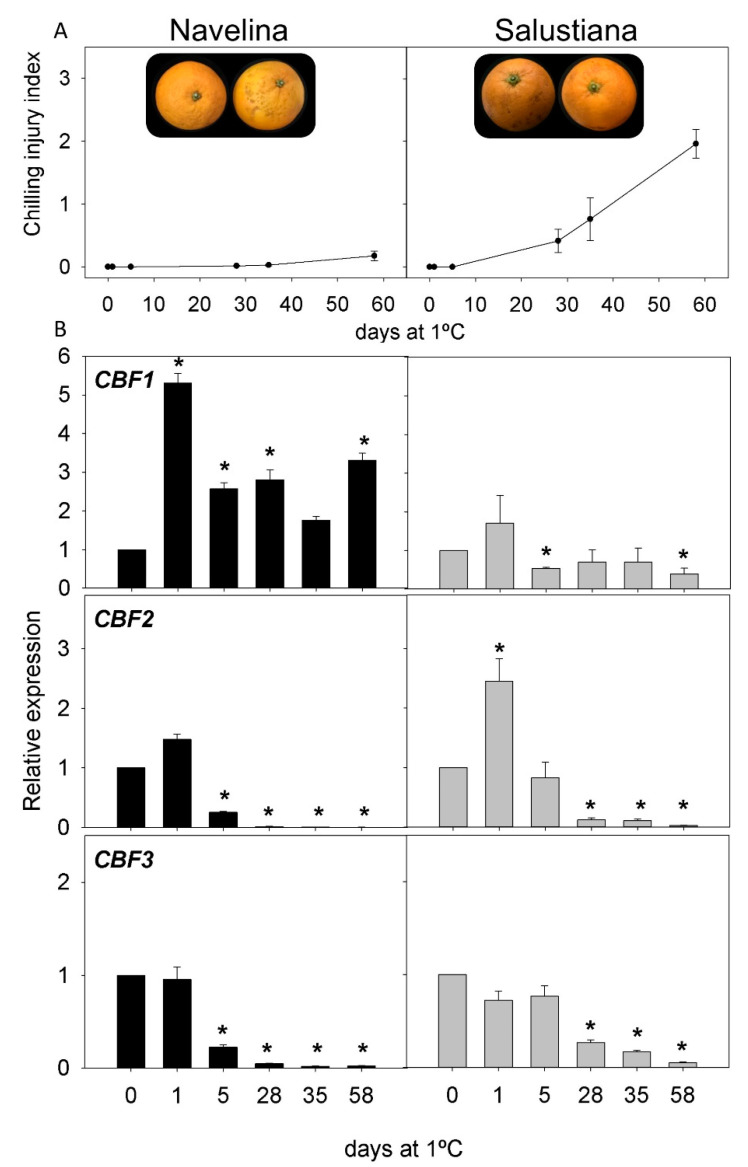
(**A**) CI index in Navelina and Salustiana oranges at harvest and during cold storage at 1 °C and (**B**) relative expression of *CBF1*, *CBF2*, and *CBF3* in Navelina (black bars) and Salustiana (grey bars) during cold storage (means ± S.E.). Pictures show the external appearance of fruit at 58 d of cold storage. For each cultivar, asterisks indicate significant differences in the expression of a *CBF* gene between each time-point and the harvest time (which were set to 1), by a Student’s *t*-test (*p* < 0.05).

**Figure 6:**
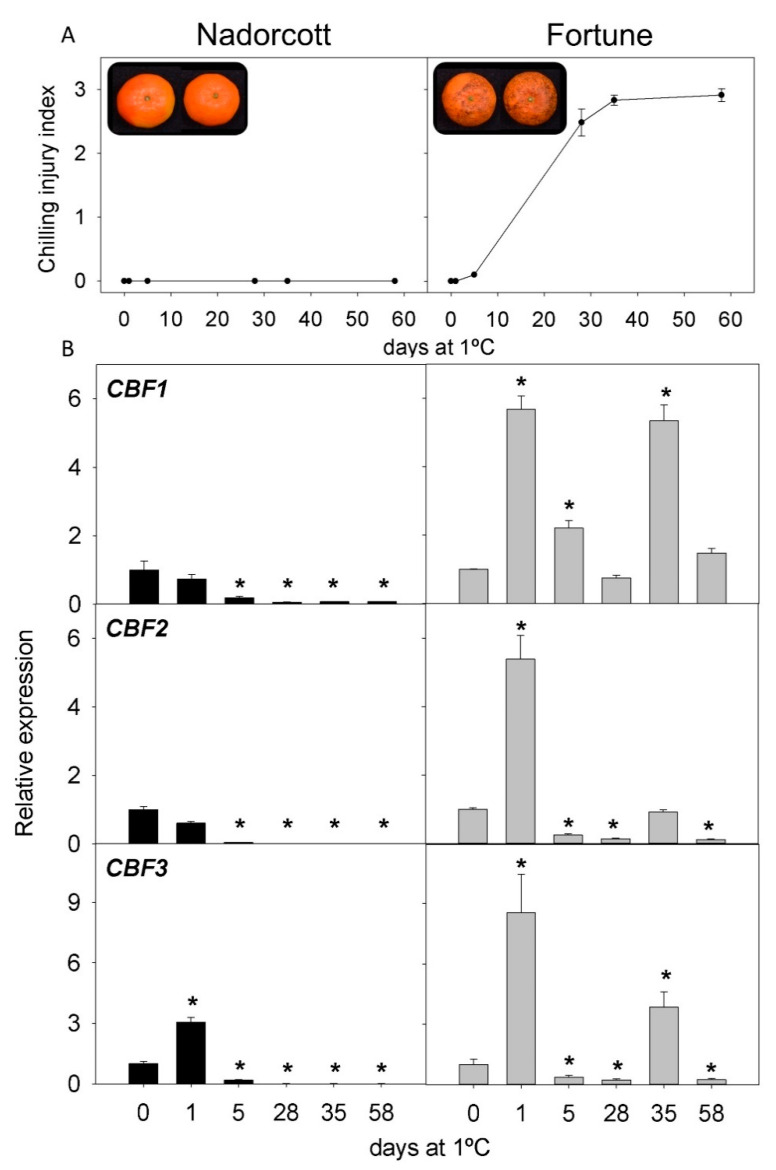
(**A**) CI index in Nadorcott and Fortune mandarins at harvest and during cold at 1 °C and (**B**) relative expression of *CBF1*, *CBF2*, and *CBF3* in Nadorcott (black bars) and Fortune (grey bars) during cold storage (means ± S.E.). Pictures show the external appearance of fruit at 58 d of cold storage. For each cultivar, asterisks indicate significant differences in the expression of a *CBF* gene between each time-point and the harvest time (which were set to 1), by a Student’s *t*-test (*p* < 0.05).

CI incidence was evaluated in the fruit of two cultivars of the most important *Citrus* species: lemons, grapefruit, oranges, and mandarins. Both cultivars showed contrasting sensitivity to CI during storage at 1 °C for two months (Figure 3, Figure 4, Figure 5 and Figure 6). In lemons, the fruit of Lisbon were more resistant to CI than those of Meyer, since after 58 d of storage CI, the index in Lisbon was about 0.28, whereas in Meyer, it was 2.5 (Figure 3A). Initial CI symptoms appeared in the peel of Meyer after 14 d of cold exposure, showing brown depressed areas that progressively increased in extension and developed large clustered brown areas. By contrast, the fruit of the Lisbon cultivar only developed small scattered pits on the fruit surface (Figure 3A). Grapefruit cultivars also showed differences in CI incidence, with lower levels in Star Ruby than in Marsh fruit: 1.57 and 2.81 after 58 d of storage, respectively. Marsh symptoms appeared as early as 14 d after storage (Figure 4A). Sweet orange fruits also showed contrasting sensitivity to CI, Navelina being more tolerant than Salustiana (CI index of 0.17 and 1.96, respectively, at the end of the storage period). CI symptoms in orange fruit developed as discrete sunken areas that progressively became bronze, covering a wide surface of the fruit (Figure 5A). A comparison of CI between the fruit of Fortune and Nadorcott mandarins also revealed marked differences in susceptibility to CI. Fortune fruit were very susceptible to CI (2.90 after 58 d), while Nadorcott fruit were highly tolerant during the whole storage period (0.26 after 58 d). The onset of chilling symptoms in Fortune was detected after 21 d of storage and manifested as the typical pitting symptoms speared over the fruit surface, whereas Nadorcott mandarins were almost devoid of damage (Figure 6A).

The expression of the three *CBF* genes (*CBF1*, *CBF2*, and *CBF3*) in the flavedo of the fruit of the eight *Citrus* cultivars was evaluated during 58 d of cold storage. Lisbon lemon showed an early induction of *CBF1* after cold exposure (1 and 5 d), decreasing afterward (except for 35 d), whereas, in the Meyer cultivar, its expression decreased. The expression of *CBF2* and *CBF3* decreased in Lisbon, especially at 1 d, 28 d, and 58 d, while Meyer showed a transient increase in *CBF3* after 5 d of cold storage (Figure 3B). In Star Ruby grapefruit, expression of the three *CBF* genes increased after 1 and 5 d, *CBF1* being much higher than that of *CBF2* and *CBF3* (10, 1.5, and 2-times higher than the initial, respectively). In contrast, the expression in Marsh grapefruit remained almost unchanged during cold storage for *CBF1* but decreased in *CBF2* and *CBF3* (Figure 4B). In Navelina orange, transcript accumulation of *CBF1* showed a transient increase (×5.5) after 1 d of cold storage, decreasing afterward, whereas, in the cold-sensitive Salustiana, it experienced minor alterations (Figure 5B). *CBF2* mRNA abundance increased in Salustiana orange after 1 d of cold storage, and *CBF3* displayed a similar accumulation in both cultivars (Figure 5B). In the fruit of the CI-sensitive Fortune mandarin, an early (1 d) and a sharp increase in the expression of the three *CBFs* was observed that decreased afterward to peak again after 35 d. Interestingly, in the CI-tolerant Nadorcott mandarin, the expression of the three *CBFs* did not experience important changes during the whole storage period (with the exception of *CBF3* at 1 d) and the corresponding mRNAs accumulated to lower levels than in the sensitive mandarin (Figure 6B).

### 2.3. Effect of Light Deprivation on CI and Expression of CBF Genes in Fruit of Cold-Sensitive Citrus Fruits during Cold Storage

Previous studies have shown that light deprivation induced tolerance to CI during postharvest storage of citrus fruit of sensitive cultivars [9]. To further investigate the involvement of *CBF* genes in the tolerance of citrus fruit to CI, the fruit of the cold-sensitive cultivars Meyer, Marsh, Salustiana, and Fortune, were covered on the tree, and their responses to cold storage and the expression of CFB genes were evaluated.

**Figure 7:**
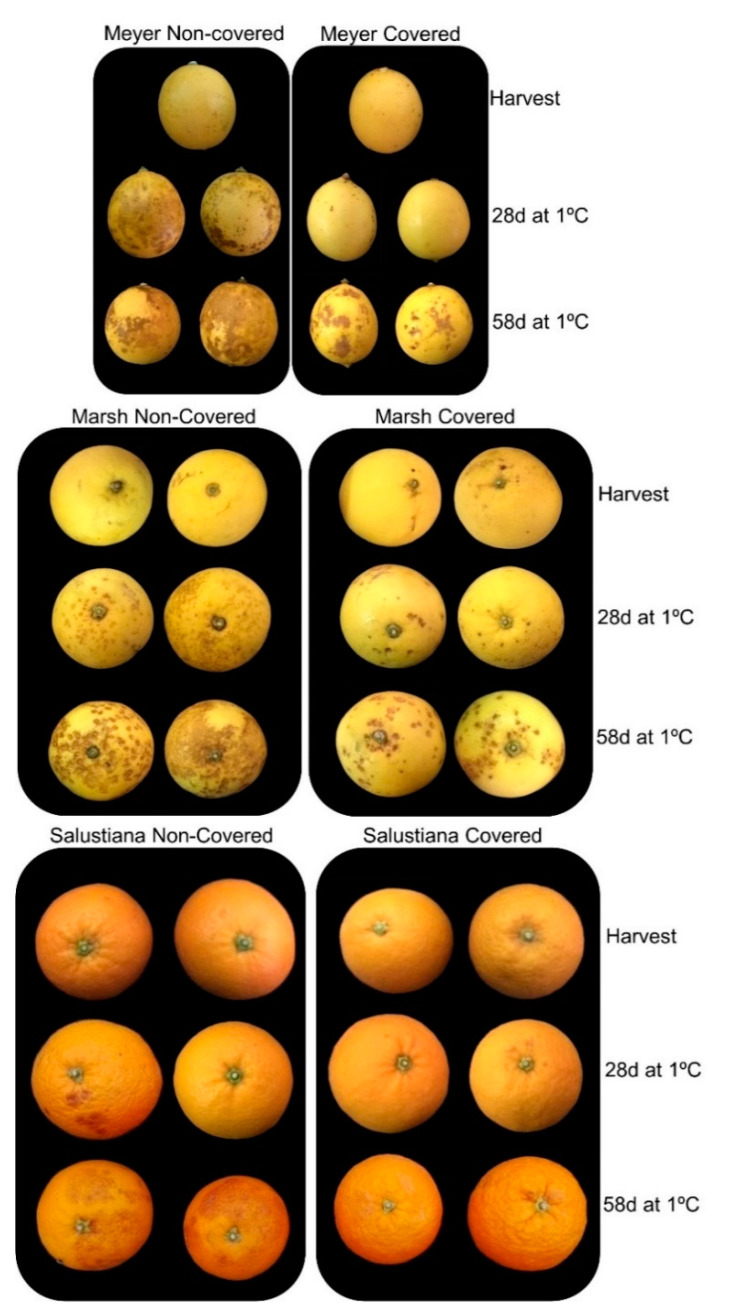
CI symptoms in non-covered and covered fruit of Meyer lemon, Marsh grapefruit, and Salustiana orange at harvest and after 28 and 58 d of cold storage at 1 ± 0.5 °C.

**Figure 8:**
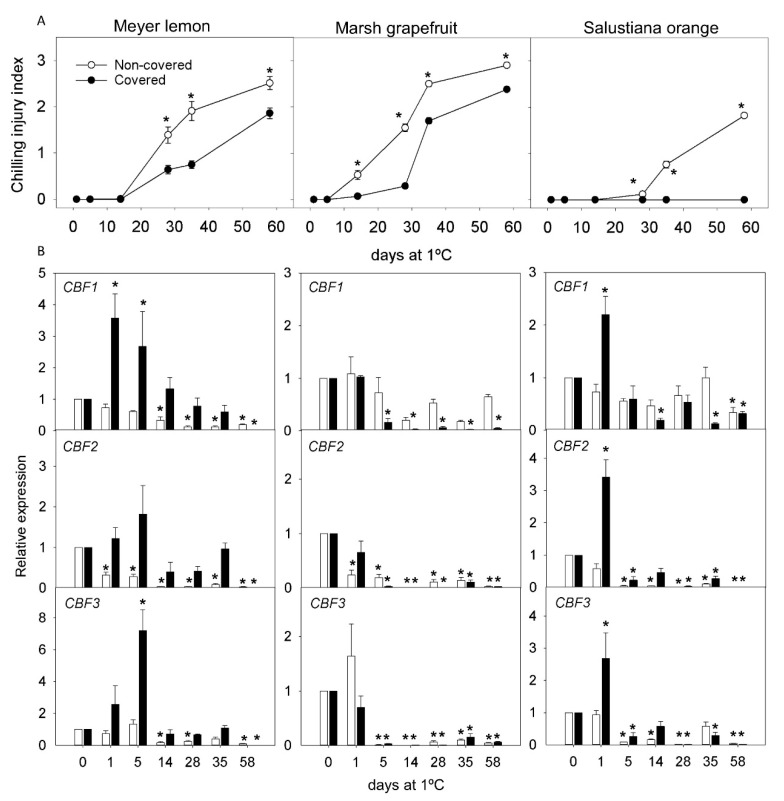
(**A**) CI index in non-covered and covered Meyer lemon, Marsh grapefruit, and Salustiana orange during cold storage at 1 °C and (**B**) relative expression of *CBF1*, *CBF2*, and *CBF3* in non-covered (white bars) and covered (black bars) fruit of Meyer lemon, Marsh grapefruit, and Salustiana orange during cold storage (means ± S.E.). For each cultivar, reference samples at harvest (0) were set to 1, and asterisks indicate significant differences in CI index between non-covered and covered fruit, and for *CBF* gene expression between each time-point and harvest by a Student’s *t*-test (*p* < 0.05).

Fruit covering produced variable effects on peel color of the cultivars analyzed at harvest, with Meyer covered fruit being pale yellow in color compared to non-covered, while no differences were detected in Marsh and Salustiana. During cold storage, the color remained almost unaltered (Table S3). In general, fruit covering delayed the development and reduced the incidence of CI after 2 months of storage in Meyer lemon and Marsh grapefruit. The most remarkable effect of fruit coverage was the virtual absence of CI symptoms in the fruit of Salustiana oranges (Figure 7 and Figure 8A). In the fruit of the CI-sensitive Fortune mandarin, light deprivation only delayed the rate of CI, but at the end of storage, the CI index was similar between covered and non-covered fruit (data not shown). Therefore, Fortune mandarin was discarded for further analysis of *CBF* gene expression.

Analysis of the expression profile of the three *CBF* genes revealed differences in covered and non-covered fruit of the three species in response to low-temperature storage. In lemon Meyer, fruit coverage induced an early (1 or 5 d) and transient stimulation of the expression of *CBF1* and *CBF3*, which was not observed in non-covered fruit. In Salustiana oranges, fruit coverage also induced an early (1 d) and transient increase in the expression of *CBF2* and *CBF3* genes. In Marsh grapefruit, however, the accumulation pattern of the three mRNAs was in general similar in covered and in non-covered fruit throughout the whole storage period (Figure 8).

## 3. Discussion

The family members of the CBF transcription factors were shown to play an important regulatory role in the complex molecular responses of plant tissues to cold acclimation [15,22,44,51,52]. In different plant species, it has been demonstrated that genetic manipulation of *CBF* genes increases plant tolerance and survival to low temperatures. However, in the responses of plant tissues to cold stress, both CBF-dependent and CBF-independent signaling pathways operate [53,54]. In *Citrus*, *CBF* genes from cold-sensitive and cold-tolerant genotypes have been characterized, and their potential function in the acclimation of vegetative tissue to the low temperature suggested (15). Nonetheless, the involvement of CBF-dependent pathways in the natural or induced-tolerance of citrus fruit to low-temperature stress during postharvest storage is still unknown.

With the aim of exploring the role of *Citrus* CBFs in the fruit response to cold, we performed an in silico search to identify all potential members of this family in *Citrus*. Based on sequence homologies with *Arabidopsis* CBFs, in *Citrus*, this family is composed of three genes: *CBF1*, *2*, and *3*. The sequences analysis showed that the *Citrus* CBFs contained most of the characteristic motifs described for this subgroup of transcriptions factors, which distinguishes them from the other AP2/ERF family members [40,45,47]. However, the motifs were not fully conserved in all *Citrus* CBFs (Figure 1), which may affect their binding to target genes or determine their specificity as has been suggested for different CBF members in other plant species.

Different studies reported increased expression of *CBFs* in fruit, such as peach [28,55], mango [56], Hami melon [38], oil palm fruit [24], grape [40,41], and tomato [37,57,58] subjected to low temperature but also other stresses or ABA (abscisic acid) treatment, indicating a regulation by osmotic-related stress. Interestingly, in several instances, increases in their expression have been associated with a higher tolerance to chilling-related disorders [28]. In contrast, in Cardinal table grapes, *VvCBF1* and *VvCBF4/VviDREBA1–1*, were induced by CO_2_ treatment but not by cold storage [41], revealing possible CBF-independent mechanisms in the response of the berries to low temperature.

In the current work, we took advantage of the natural diversity in the susceptibility of the fruit from different *Citrus* species and cultivars to develop CI during postharvest cold storage, to explore whether CBF transcription factors are involved in this natural cold tolerance. Cold stress during postharvest storage is manifested in citrus fruit by a series of morphological and structural alterations in the peel (pitting, browning, and staining), referred to as chilling injury that diminishes the external and commercial quality of the fruit, causing consumer rejection and economic losses. The genetic variability in the tolerance to postharvest CI has been recognized for a long time, and among the main *Citrus* species cultivated worldwide, lemons and grapefruit are more susceptible than oranges and mandarins [59]. Interestingly, within each main *Citrus* species, there are also wide varietal differences in the tolerance to develop CI, indicating the influence of genetic factors in the fruit tolerance to postharvest CI [2,59]. Our results show marked differences in the CI incidence between the two cultivars of lemons, grapefruit, oranges, and mandarins used in this study (Figure 3, Figure 4, Figure 5 and Figure 6) that are consistent with previous works. The difference in the rate of CI development between Lisbon and Meyer lemon is remarkable (Figure 3). Genetic evidence has revealed a different phylogenetic origin of both lemons (*C. limon* and *C. meyeri*), and it is likely that the divergent response to cold stress is related to the different genetic background of both genotypes [60]. Meyer is highly susceptible to cold, developing CI symptoms as early as two weeks of cold storage.

Among grapefruit (*C. paradisi*), it is already documented that the red Star Ruby is less sensitive to CI than Marsh, and its tolerance is related to the accumulation of the antioxidant lycopene in the peel [10,11]. Similarly, Salustiana oranges (*C. sinensis*) were more prone than Navelina oranges to develop CI upon cold storage (Figure 5). Fortune mandarin is a hybrid of Dancy mandarin x Clementine mandarin recognized by the high susceptibility to develop CI, whereas Nadorcott is a mandarin derived from Murcott mandarin, which is resistant to CI (Figure 6) [2]. Together, these results reinforce the notion that the genetic background of the cultivar and species is a major factor prevailing in the susceptibility of citrus fruit to CI [2].

A comparison of the changes in the expression of the three *CBFs* genes (*CBF1*, *CBF2*, and *CBF3*) in the fruit of sensitive and tolerant cultivars of *Citrus* revealed potential participation of each member in the tolerance/sensitivity to CI during storage. Table 1 summarizes the comparative changes in these processes in lemon, grapefruit, and orange cultivars in covered and non-covered fruit. The expression of the genes showed major changes at the early stages of cold exposure (1–5 d), as may be explained by the role of these transcription factors in the regulation of other cold-induced responses [53]. Interestingly, *CBF1* was the most induced gene in the peel of CI-tolerant cultivars (Lisbon lemon, Star Ruby grapefruit, and Navelina orange). This activation occurred between 1 and 5 d after initiation of the cold exposure, whereas in Star Ruby grapefruit, it declined after 1 month of storage (Figure 4). In Navelina orange, it remained at high levels (Figure 5), and an intermediate response was found in Lisbon (Figure 3). With the exception of the chilling-sensitive Fortune mandarin, in which *CBF1* was stimulated after 1 d of cold storage (Figure 6), the expression of this transcription factor remained virtually unaltered in the fruit of the lemons, grapefruit, and oranges sensitive to CI (Table 1). These results suggest a positive association of *CBF1* expression with cold tolerance in *Citrus* cultivars, with the exception of mandarins. Although from these results, the function of this transcription factor on fruit responses to cold cannot be delineated, it appears that CBF-mediated responses may exist in the tolerance of citrus fruit to cold stress. Results suggest a long-term modulation of CBFs expression after cold exposure in citrus fruit, showing a sustained response several days after initiation of the stress stimuli.

Regarding the potential role of *Citrus* CBF1 in fruit cold-stress tolerance, it is worth mentioning that phylogenetic analysis and conservation of motifs among the *Citrus* CBFs suggest that CBF1 is more similar to other plant CBFs involved in cold stress responses than CBF2 and 3 (Figure 2). The DREBA1 signature sequence PKKPAGR upstream the AP2 domain and the C-terminal LSWY motif are only fully conserved in CBF1 (Figure 1 and Figure S1). Deletions or mutations in the PKKPAGR sequence of *Arabidopsis* CBF1 significantly affect its ability to induce expression of target cold-regulated genes [61], and the LWSY motif, only identified in CBF1, is conserved at the end of the C-terminal of most of the DREB1-type proteins [40,46], suggesting an important role in the function of these DREB1-type proteins. Thus, alterations in key amino acids or the absence of CBF characteristic motifs in *Citrus* CBF2 and 3 may impair their functionality to the cold response.

In other plants, *CBF1* was also described as a key cold response regulator, as in *Arabidopsis*, where its expression was drastically reduced in mutants with impaired ability to cold acclimation. These mutants also showed a modest reduction in *CBF3* and *CBF2* expression during cold exposure, suggesting the involvement of the three genes in *Arabidopsis* cold acclimation [51]. Similar to our results, *CBF1* expression has been associated with chilling-resistant table grape cultivars induced by CO_2_-treatments that alleviated cold stress [40,41], revealing a diverse function of these genes depending on the fruit species, developmental stage, and severity of the cold stress [58]. In Hami melon fruit, an increase in *CBF1* and *CBF3* gene expression during cold storage was correlated with a lower CI index in the tolerant cultivar [38]. During tomato fruit storage at 2.5 °C, *CBF1* expression peaked after 1–24 h of cold exposure and declined afterward [58]. In peach fruit, *PpCBF* genes induction was accompanied by a decrease in CI symptoms during postharvest cold storage [28]. In tomato fruit, an increase in *SlCBF1* expression was observed after 1 to 24 h of cold storage (2 °C) [58] and to be responsive to cold and exogenous ethylene [37]. Moreover, a lower CI incidence during postharvest storage was registered after nitric oxide inductor treatment in tomato fruit, showing a fast (30 min) increase in the expression of *SlCBF1* [57].

Based on our results, the expression of *Citrus CBF2* or *CBF3* genes in CI-tolerant and CI-sensitive fruit did not reveal a clear relationship with the susceptibility to CI. Transcriptional changes on both genes were not as remarkable as those of *CBF1*, and the changes observed were not consistent with their involvement in cold tolerance or cold sensitivity (Table 1). *CBF2* and *CBF3* appear not to play a key function in the response of citrus fruit to cold tolerance of sensitivity.

Light deprivation, by shading the fruit on the tree during the last 3 months of development, had a protective role, reducing CI during postharvest cold storage in the sensitive cultivars of lemons, grapefruit, and oranges (Figure 6 and Figure 7). The induction of CI-tolerance by fruit shading has been previously described in the fruit of the red Star Ruby grapefruit and associated with an increase in carotenoid and lycopene content in the peel and enhanced antioxidant capacity [10,11]. Light deprivation in yellow-colored fruit of Meyer lemon and Marsh grapefruit and Salustiana oranges only produced slight modifications in the color of the peel, compared to light-exposed fruit (Table S3). The tolerance to CI induced by fruit covering, however, was not uniform when comparing the three cultivars studied. Salustiana orange cultivar had a major reduction in CI (Figure 7). Fruit covering has been shown to affect other metabolic pathways and metabolites in citrus fruit, such as ascorbic acid or carbohydrates [62,63]. These results suggest that the response of yellow-colored citrus fruit to light deprivation in carotenoid content and composition, and CI may be different to that of orange-colored fruit, as Salustiana oranges, and that other biochemical and molecular factors may be implicated in the induction of tolerance to CI.

Interestingly, the tolerance to CI induced by shading in Meyer lemon and Salustiana oranges was associated with a rapid and transient enhancement of the expression of at least two of the three *CBF* genes, which did not occur in non-covered fruit (Figure 8). These results reinforce the previous notion that *CBF1* appears to be associated with the induction of cold tolerance in sensitive cultivars (except for mandarins). Whether the increased expression of *CBF2* and *CBF3* reflect their involvement in the acquisition of cold tolerance or may be a response to light deprivation during the last phases of development remains to be determined. Together, these results suggest that CBFs-dependent pathways mediate at least part of the induction of cold tolerance in sensitive *Citrus* cultivars. Although these responses may be cultivar-specific, our data suggest that *CBF1* participates in the natural tolerance of sensitive cultivars to CI and that it is favored by fruit shading in Meyer lemon and Salustiana oranges.

## 4. Materials and Methods

### 4.1. Plant Material, Preharvest Treatments, and Storage Conditions

Fruit of the following cultivars of the main Citrus species: grapefruit (*Citrus paradisi* cv. Star Ruby and cv. Marsh), lemon (*Citrus limon* cv. Lisbon and *Citrus meyeri* cv. Meyer), orange (*Citrus sinensis* cvs. Salustiana, Washington Navel and Navelina), and mandarin (*Citrus reticulata* cv. Fortune and cv. Nadorcott), with contrasting sensitivity to cold damage were used in this study. Fruits were harvested at full maturity from commercial orchards located in Salto, Uruguay (grapefruit, lemon, and orange cultivars) and Valencia, Spain (mandarin cultivars). Trees were grown under standard agronomical conditions.

To evaluate the effect of light exposure on CI and gene expression, the fruit of the different cultivars were covered with black plastic bags (leaving the bottom-end open to allow gas exchange) at immature green stages around three months before harvest as previously described [9,62]. Control (non-covered) fruit were located outside of the tree canopy and exposed to normal photoperiod conditions. Fruit of both treatments were harvested at commercial maturity, selected for uniformity and free of any defect or damage, and stored at 1 ± 0.5 °C for up to 58 d. During cold storage, the CI incidence was periodically evaluated. Flavedo tissue was excised, frozen in liquid nitrogen, ground to a fine powder, and stored at −80 °C until RNA extraction. Comparison of CI susceptibility between varieties was conducted in two consecutive seasons (2017 and 2018), and a comparison of the postharvest performance in covered and non-covered fruits was conducted in independent experiments in different seasons (2018 and 2019), representing the average of both seasons.

### 4.2. Fruit Color and Chilling Injury Evaluation

At harvest and during storage, peel color of whole fruit was measured using a Minolta CR-400 colorimeter (Minolta, USA) on three areas of the equatorial plane of the fruit and expressed as the ICC (citrus color index), calculated with Formula (1). A lower ICC value (more negative) represents green fruit, near-zero values correspond to yellow fruit at the color break, and orange- to red-colored fruit reflects positive values.

ICC = (1000 × a)/(L × b),(1)

Fruit were inspected for CI symptoms (intensity and extension of the damage) after 1, 5, 14, 28, 35, and 58 d in cold storage. The severity of the symptoms was assessed visually using the following scale: 0 = no pitting; 1 = pitting covering <25% of the fruit surface; 2 = pitting covering between 25 and 50% of the surface; 3 = 50–100%. CI index was calculated using the Formula (2) described in [10]:

(2) $$\mathrm{CI}\;\mathrm{index}=\frac{\sum \left[\left(\mathrm{CI}\;\mathrm{level}\right)\times \left(\mathrm{Number}\;\mathrm{of}\;\mathrm{fruits}\;\mathrm{at}\;\mathrm{the}\;\mathrm{CI}\;\mathrm{level}\right)\right]}{\mathrm{Total}\;\mathrm{number}\;\mathrm{of}\;\mathrm{fruits}\;\mathrm{evaluated}}$$

The experimental design was completely randomized, and results correspond to the mean ± S.E. of four replicates of 20 fruit each.

### 4.3. RNA Extraction and Quantitative Real-Time PCR Analysis

Total RNA was isolated from plant material following the protocol described in [64] with modifications. Two tubes of 2 mL each with 0.2 g of flavedo tissue were processed by sample. 770 µL of extraction buffer (200 mM Tris-HCl, pH 8.0, 400 mM NaCl, 50 mM Na_2_EDTA, 2% (w/v) Sarkosyl, 1% (w/v) poly(vinylpyrrolidone), and 1% (v/v) β-mercaptoethanol), and 380 µL of phenol was added to each tube. Tubes were vortexed and incubated at 65 °C for 15 min. Three hundred and eighty microliters of chloroform:isoamilic alcohol (24:1) were added to each tube and centrifuged at 4000× *g*, 10 min at room temperature. The aqueous phase was transferred to a new 2 mL tube and reextracted with 380 µL of phenol and 380 μL of chloroform:isoamilic alcohol (24:1). Tubes were centrifuged at 4000× *g* for 10 min, the aqueous phase was transferred to a new 1.5 mL tube, and RNA was precipitated with 1.5 vol of ethanol. After precipitation, tubes were centrifuged at 20,000× *g* for 30 min at 4 °C. The pellet was washed with 500 µL of 70% ethanol and resuspended with 300 µL of ultrapure RNAse-free water. Replicate tubes from the same sample were mixed, 1/3 vol of LiCl 12 M was added, and tubes were incubated on ice at 4 °C overnight. Tubes were centrifuged at 20,000× *g* for 30 min at 4 °C, and the pellet was washed with 800 µL of 70% ethanol. The pellet was dried at room temperature and resuspended in 50 µL of ultrapure RNAse-free water. RNA was quantified, with a recording absorbance at 260 nm, and quality was verified by sample absorbance at 260/280 nm and 260/230 nm. The integrity of RNA was evaluated by 1% agarose gel electrophoresis.

For each sample, 10 µg of RNA was treated with a DNAse Turbo DNA-free^TM^ kit (ThermoFisher Scientific, Lithuania) according to the manufacturer’s instructions. After DNAse treatment, cDNA synthesis was performed with 1 µg of treated RNA and using a RevertAid Reverse transcriptase kit (Thermo Scientific, Lithuania) according to the manufacturer’s specifications.

Quantitative RT-PCR reactions were performed using an Applied Biosystems StepOne^TM^ Plus Real-Time PCR System (Applied Biosystems, San Francisco, CA, USA). Each reaction consisted of 2 µL of a dilution 1:4 of cDNA, 1 µL of primer mix (10 µM each), and 10 µL of SensiFAST™ SYBR^®^—HiRox kit (Bioline, UK). Primers used for amplification of *Actin*, *CBF1*, *CBF2*, and *CBF3* are listed in Table S1. The cycling condition for all genes analyzed consisted of 10 min at 95 °C for pre-incubation, 40 cycles of 15 s at 95 °C, 15 s at 59 °C, and 15 s at 72 °C. Fluorescence intensity data were acquired during the extension step.

The specificity of the PCR reaction was confirmed by the presence of a single peak in the dissociation curve performed after the amplification steps. Relative expression was determined using the Pfaffl method [65], where gene expression was normalized using the expression levels of *Actin*, a constitutive gene, in the assay conditions [66]. For all genes and cultivars analyzed, the reference sample was harvest condition. All gene expression data were represented as the mean of three replicates ± SE. Gene expression was analyzed using the Student’s *t*-test, being the difference between harvest time (0 d, which were set at 1) and each sampling time (1, 5, 28, 35, or 58 d) considered significant when *p* < 0.05 in a two-tailed analysis.

## Figures and Tables

**Table 1:** Summary of the changes in chilling injury (CI) and the relative expression levels of *CBF1*, *CBF2*, and *CBF3* in the flavedo of covered and non-covered fruit of different Citrus varieties.

		Non covered				Covered			
Specie	Variety	CI	*CBF1*	*CBF2*	*CBF3*	CI	*CBF1*	*CBF2*	*CBF3*
Lemon	Lisbon	-	++	-	-	n.d.			
	Meyer	+++	--	+	+	+	++	-	+++
Grapefruit	Star Ruby	+	+++	+	+	n.d.			
	Marsh	+++	--	-	-	++	-	-	-
Orange	Navelina	-	+++	-	-	n.d.			
	Salustiana	++	-	-	-	--	+	++	++

Symbols indicate: -, no changes or reduction; +, increases over initial; n.d., not determined.

## Data Availability

The data presented in this study are available in the article or supplementary material.

## Supplementary Materials

The following are available online at https://www.mdpi.com/1422-0067/22/2/804/s1, Table S1. qRT-PCR primer sequences; Table S2. Percent identity matrix of deduced amino acid sequences of CBFs from sweet orange, *Arabidopsis*, table grapes, and tomatoes; Table S3. Fruit color (ICC values) at harvest time and during cold storage of Meyer, Marsh, Salustiana, and Fortune non-covered and covered fruit. Figure S1. Alignment of *Citrus sinensis* CBF proteins and other plant CBFs.

## Abbreviations

ABA	Abscisic acid
AP2	Apetala2
CI	Chilling injury
CBFs	C-repeat binding factors
COR genes	Cold regulated genes
ICC	Citrus color index
phyB	Phytochrome B
PIF3	Phytochrome-interacting transcription 3
DREB	Dehydration-responsive element binding
HCA	Hydrophobic cluster analysis
