# Supplementary material for: Transcriptional Analysis of C-Repeat Binding Factors in Fruit of Citrus Species with Differential Sensitivity to Chilling Injury during Postharvest Storage
Source: Int J Mol Sci. 2021 Jan 15;22(2):804. doi: 10.3390/ijms22020804 (PMC7830921; doi:10.3390/ijms22020804)

Table S1. Primers used for relative gene expression in citrus fruit.

Gene	Primer	Sequence (5’-3’)
Actin	*Act-F*	TTAACCCCAAGGCCAACAGA
	Act-R	TCCCTCATAGATTGGTACAGTATGAGAC
CBF1	*CBF1-F*	CCTTATCATCTCCGTGTTCGCGTT
	CBF1-R	CTCTTAACTGGCTGTGTGTGTGAAATCT
CBF2	*CBF2-F*	AGGAGAGTGTTTAAGGAGACACGTCA
	CBF2-R	GCAGCCATTTCTGGAGAAGGATAGG
CBF3	*CBF3-F*	CAGAAGCATTCAGGCTGTCCC
	CBF3-R	CTTCGTATCATCATCATCATACTCACA

Table S2. Percent identity matrix of deduced amino acid sequences of CBFs from sweet orange (this work). Arabidopsis [15]. grape [40] and tomato [22].The identity matrix was created using CLUSTAL Omega (https://www.ebi.ac.uk/Tools/msa/clustalo/).

	1	2	3	4	5	6	7	8	9	10	11	12
**1: CitrusCBF1**	100.00	60.77	59.47	62.31	60.89	61.88	50.99	49.28	65.53	60.61	62.56	67.01
**2: CitrusCBF2**	60.77	100.00	72.82	53.85	50.24	52.61	46.86	43.84	56.34	54.63	53.74	58.50
**3: CitrusCBF3**	59.47	72.82	100.00	57.45	53.40	54.45	50.27	50.79	62.90	56.42	56.68	57.87
4: AtCBF1	62.31	53.85	57.45	100.00	87.79	88.21	46.57	49.28	61.62	59.90	57.00	61.17
5: AtCBF2	60.89	50.24	53.40	87.79	100.00	87.44	44.66	45.28	58.21	58.25	55.67	60.53
6: AtCBF3	61.88	52.61	54.45	88.21	87.44	100.00	46.60	46.23	62.19	62.69	58.13	61.90
7: VvDREBA1-6	50.99	46.86	50.27	46.57	44.66	46.60	100.00	63.03	49.25	52.33	51.52	51.32
8: VvDREBA1-7	49.28	43.84	50.79	49.28	45.28	46.23	63.03	100.00	47.29	52.31	50.49	49.74
9: VvDREBA1-1	65.53	56.34	62.90	61.62	58.21	62.19	49.25	47.29	100.00	60.29	58.96	65.69
10: SlCBF1	60.61	54.63	56.42	59.90	58.25	62.69	52.33	52.31	60.29	100.00	83.73	72.28
11: SlCBF2	62.56	53.74	56.68	57.00	55.67	58.13	51.52	50.49	58.96	83.73	100.00	72.28
12: SlCBF3	67.01	58.50	57.87	61.17	60.53	61.90	51.32	49.74	65.69	72.28	72.28	100.00

Table S3. Fruit color (ICC values) at harvest time and during cold storage of Meyer, Marsh, Salustiana and Fortune non-covered and covered fruit.

*Means of non-covered and covered fruit from each citrus cultivar followed by different letters showed significant differences (Tukey $p<0.05$).

Treatment	Harvest	1d	5d	14d	28d	35d	58d
Meyer non-covered	-0.28 a	0.06 a	-0.43	-0.44 a	-0.34	-0.51	-0.38
Meyer covered	-0.67 b	-0.54 b	-0.68	-1.06 b	-0.55	-0.21	-0.62
Marsh non-covered	-0.99	-0.99	-0.97	-0.66	-0.74	-0.96	-1.01
Marsh covered	-0.66	-0.88	-0.80	-0.83	-0.86	-0.76	-0.71
Salustiana non-covered	4.20	4.89	3.83	4.54	3.68	4.56	4.43
Salustiana covered	4.20	4.07	4.30	4.47	4.31	4.86	4.07

Figure S1. Alignment of *Citrus sinensis* CBF proteins and other plant CBFs .The alignment of CBF proteins was created using CLUSTAL W program. Numbers on the right denote the number of amino acid residue. Residues identical for all the sequences in a given position are in white text on a black background and 75 to 100% homologue residues are presented on grey background. The sequences used to generate the alignment and their accession numbers are as follows: : *Citrus* CBF1 (orange1.1g028094m). CBF2 (orange1.1g026103m) and CBF3 (orange1.1g029015m) ; *Arabidopsis thaliana* AtCBF1 (AT4G25490.1). AtCBF2 (AT4G25470.1) and AtCBF3 (AT4G25480.1); *Solanum lycopersicum* SlCBF1 (Q8S9N5). SlCBF2 (XP_004234350.1) and SlCBF3 (AAS77819.1); *Vitis vinifera* VviDREBA1-6 (MF445008). VviDREBA1-7 (MF445009) and VviDREBA1-1 (MF445007). The CBF characteristic motifs : PEST motif (grey). PKKPAGR motif (pink). AP2 domain (yellow). DSAWRL motif (blue). A(A/V)xxA(A/V)xxF motif (green). LWSY motif (red) and the C-terminus hydrophobic clusters (black). are marked in citrus CBF1. The conserved motifs of AP2 domain (YRG. WLG. and RAHD. and V14 and E19) and marked with asterisks.

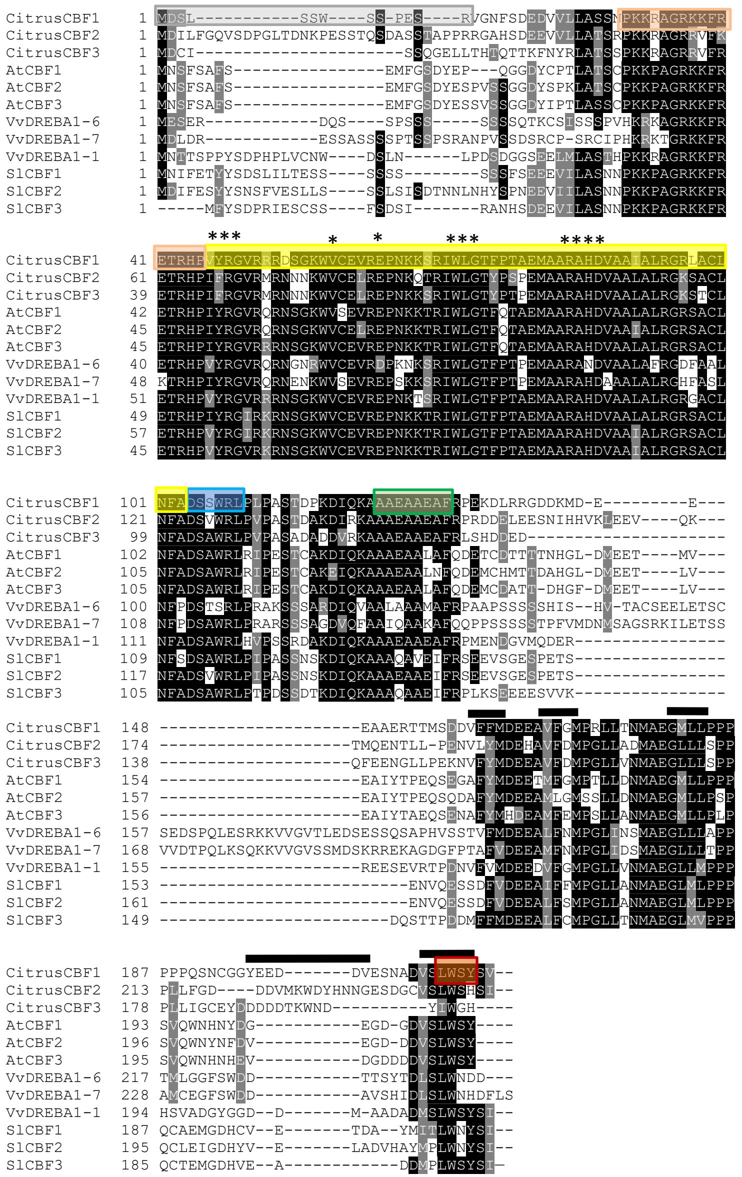

Supplement: Supplementary file 1 [file ijms-22-00804-s001.pdf]
